# Democratic Accountability in Strategic Coordination Bodies — An Investigation of Governance in Swedish Elder Care

**DOI:** 10.5334/ijic.4207

**Published:** 2019-07-09

**Authors:** Staffan Johansson, Andreas Liljegren

**Affiliations:** 1 Department of Social Work, University of Gothenburg, SE

**Keywords:** accountability, network governance, coordinated care, eldercare

## Abstract

The establishment of strategic coordination bodies with members from different agencies and that are governed by various laws and regulations can be understood as an answer to the demand for improved coordinated care for citizens with complex needs, such as frail older people. However, this demand raises fundamental questions of democratic control and accountability in the modern welfare state. Although these issues are addressed in current literature on network governance, they have not been investigated empirically very much.

The aim of this paper is to investigate coordination bodies as important actors in integrated care, and especially to investigate how the members of these governance networks perceive their own influence and how they are held accountable by their principals.

This study is conceptually built on theories of network governance and accountability. The empirical investigation is based on a survey with 545 respondents from 73 different coordination bodies in Sweden. The analysis shows that there seems to be an imbalance between perceived influence and perceived demands from different stakeholders to account for the services. This imbalance provides an opening for a discussion of how to improve the current situation for vulnerable groups and about new perspectives on accountability and power in the modern welfare state.

## Introduction

### Problem statement

The coordination problems that occur around frail older people with multi-morbidity and around other vulnerable citizens with complex needs seem to be similar in many countries, regardless of which kind of welfare policies are applied [1]. Extensive research in the area of social policy shows that the services that are provided to many groups of vulnerable citizens with complex needs require effective coordination, in part because the responsible agencies belong to different principals and are governed by different laws and regulations [2,3,4].

The Swedish government is one of many national governments that have launched policies for frail older persons with multi-morbidity, with the aim of preventing vulnerable citizens from “falling through the cracks” in the welfare system. One recent example is a comprehensive program named “Coordinated Elder Care,” which aims to create better forms of management and coordination for this target group by implementing quality records and establishing strategic coordination bodies between municipalities, counties, and representatives for private care providers. The overall aim is to enhance quality of care and quality of life for frail older people through a more integrated care between the involved care providers [5]. In Sweden, the responsibility for healthcare services belongs to the counties, whereas the responsibility for social services belongs to the municipalities. It should be noted that some of these healthcare and social service operations are run by contracted private for-profit and non-profit organizations. The roles and responsibilities of the involved organizations and agencies will be described in more details later. Nevertheless it can be noted that the program has many similarities with ideas and evidence identified in previous research [see 6,7].

The establishment of strategic coordination bodies—which are network organizations—with members from municipalities, counties, and private organizations can be understood as an example of “horizontal management” [8], or as a development “from government to governance” [9,10,11]. Such organizational arrangements are in line with previous research on effective coordination, but raise fundamental questions of democratic control and accountability of the modern welfare state. An important prerequisite for democratic governance is that there must be opportunities to keep responsible actors *accountable* to their principals [12]. However, the parliamentary governance chain model is difficult to apply to complex welfare activities that contain many organizations and professions, along with their interactions [13]. Therefore, an important question is whether the use of networks by the state and by public authorities for the purposes of policy formulation, implementation, and delivery can be considered an instrument of *democratic* governance. One such complex welfare activity is the area of care for older persons with complex healthcare and social service needs.

The issue of democratic governance and accountability can also be a matter of *effective* governance and provision of high quality care for vulnerable groups. There is a body of research on network effectiveness that indicates that the degree of centrality and external control seems to increase the effectiveness of the network and also the quality of care for vulnerable persons who need coordinated care [see e.g. 14,15]. Network organizations that experience a clearer overall responsibility for a distinct group of vulnerable people vis-à-vis potential external reviewers could also contribute to greater clarity in the political accountablity of these groups of people and thus also increase democratic control over the care system. Hence, democratic control and accountability in governance networks seem to be important issues to investigate empirically in contemporary welfare states [16].

### The aim of this paper

The aim of this paper is to investigate coordination bodies as important actors in integrated care, and especially to investigate how the members of these governance networks perceive their own influence and how they are held accountable by their principals. In the empirical investigation we will use a model of accountability in governance networks developed by Esmark [17] that will be presented later in the paper. The following questions will be answered:

1. How are such coordination bodies staffed, and what are their tasks and responsibilities?
2. What influence do the different actor groups (i.e., politicians, civil servants, and other groups) have on the involved organizations and within the coordination bodies, and how are they held accountable by their principals (according to their own perceptions)?
3. What are the most challenging issues for such coordination bodies from a democratic point of view?

### Background – The structure and responsibilities of the elder care system in Sweden

With regard to national context, it is important to take into consideration the legal and organizational contexts in Sweden, where relatively autonomous municipalities and counties rely on a decentralized system of local self-government [18]. Of course, other agencies and organizations that are important for frail older people exist, such as pharmacies, transportation services, and civil society organizations; however, the most important link in the chain is the collaboration and coordination between the municipal social services and the healthcare provided by the counties. The municipalities and counties determine the tax rates for their citizens, but the national government guarantees that all municipalities and counties have roughly equivalent tax revenues per capita, so that they can provide about the same level of service for their residents, regardless of their residents’ ability to pay tax.

There are 290 municipalities in Sweden; these are responsible for childcare, primary and secondary education, social services, culture and leisure services, housing, water supply and sewerage, and spatial planning and rescue services. The size of the average municipality is about 35 000 inhabitants; municipality size varies from 2000 inhabitants in the smallest to 950 000 inhabitants in the largest Swedish municipality. There are 21 counties in Sweden, and these have the major task and responsibility of providing healthcare; they also have other responsibilities, such as regional communication and infrastructure. The size of the average county is about 500 000 inhabitants; county size varies from 130 000 inhabitants in the smallest to 2.3 million inhabitants in the largest Swedish county [19].

Regarding elder care, the municipalities are responsible for home care as well as care provided at nursing homes. There are social welfare committees in every municipality, which mirror the political majority in that municipality. In most cases, these positions are held by laypeople. Each municipality has some kind of social service administration with a head manager and also top managers who are responsible for typical departments, such as services directed to the disabled, to older people and to families and children in need. Although the municipalities operate most of the elder care, a fairly high proportion (about 20%) is actually provided by contracted private organizations. Older people who need municipal services must pay for the services, although the fees are scaled according to their ability to pay [20].

The counties are responsible for healthcare, which includes primary healthcare and the specialized care that is provided by hospitals. The fees for healthcare services are traditionally very low. The elected county politicians work at a greater distance from the operated services than their municipal colleagues. Administrative top managers lead the hospitals and the primary healthcare. Contracted for-profit companies provide a fairly large proportion of the primary healthcare, whereas almost all hospitals are owned and operated by the counties. For many years, there have been different kinds of coordination bodies for the care of older persons with complex needs, which have been voluntarily established by the counties and municipalities with the aim to create a more integrated care. Some of these bodies cover the county as a whole, including the municipalities located in that county. Others cover part of a county—often the catchment area of a hospital along with the municipalities located there. Some coordination bodies are staffed with politicians, others with civil servants, and still others contain both politicians and civil servants, and may also include representatives of private companies. There is also variation in the number of members of these coordination bodies: Some have just a few members, whereas others may have more than 20 members [21].

The remainder of this paper is organized as follows: Section two presents a theoretical framework and reviews some previous research on accountability issues in network governance, in order to identify crucial variables for empirical investigation, which is presented in end of the section together with methodology. The results of the empirical investigation are presented in section three. The last two sections of the paper analyze and discuss the results and then present conclusions, policy implications, and suggestions for further research.

## Theory and methods

### From local hierarchic government to community governance

Interest in collaboration, coordination, and networking organization has grown in the public administration literature [22,23,24,25]. Along with the increased demands on society to manage and solve complex societal problems, interest has increased among scholars in developing concepts and theories that can help us understand underlying mechanisms and identify possible solutions and to enhance effectiveness and quality of care. Some examples of problems that have been addressed within elder care have been investigated by Wodchis et al. [26]. Provan and Milward’s seminal study on networks for the mentally disabled may also be relevant for elder care. They compared the effectiveness of different community mental health systems in four different American cities, where outcome data indicated that network effectiveness is enhanced when the network is integrated through centralization, and when mechanisms of external control are direct and not fragmented. Moreover, network effectiveness is enhanced under conditions of system stability and also if the network is embedded in a resource-rich environment [27]. Provan and Milward’s theory of network effectiveness has been validated in many empirical studies [28]. These studies have, however, not investigated issues related to democratic accountability.

As stated above, the establishment of strategic coordination bodies in elder care can be understood as an example of the development “from government to governance.” But how can this literature help us to understand the phenomenon of “coordination bodies”? Sørensen and Torfing [29] provide a comprehensive overview and framework of the research on governance networks and have defined the concept of a governance network as follows:

(1) A relatively stable horizontal articulation of interdependent, but operationally autonomous actors; (2) who interact through negotiations; (3) which take place within a regulative, normative, cognitive and imaginary framework; (4) that is self-regulating within limits set by external agencies; and (5) which contributes to the production of public purpose [30].

This definition fits the object of study in this paper — that is, strategic coordination bodies with the aim of creating integrated healthcare and social care for older people with complex needs. Regarding the first part of the definition, stability signifies a certain degree of formality and duration; in addition, the concept of “interdependent but autonomous” actors is valid for our object of study. The subsequent parts of the definition concerning negotiation frameworks, self-regulating aspects, and the aim to contribute to public purpose, are also valid for the object of interest in this study.

### Accountability in governance network

According to Olsen [31] accountability is a principle for organizing relations between rulers and those who are ruled, and for making public officials accountable to citizens. Within highly institutionalized regimes, accountability has often become routine. Power relations and expectations of how accountability can be achieved are taken for granted, which means that the “sleeping bear” is sleeping most of the time. There is widespread agreement about who is accountable to whom, for what, under what circumstances, and according to which normative criteria. It should be clear who should be blamed if things go wrong [32].

But what about democratic accountability of *governance networks*? Seen from the perspective of traditional theories of liberal democracy, governance networks represent a threat to democracy because they undermine the borderline between state and society; from this perspective, governance networks might be effective, but they are certainly not democratic. However, according to Sørensen and Torfing [33], governance networks can indeed be perceived as democratic—from a post-liberal point of view—due to their ability to solve policy problems experienced by the people more effectively than alternative institutional arrangements. Governance networks may also be an important means of establishing linkages and bridges between stakeholders with different points of identification, in order to enhance communication, coordination, negotiation, and cooperation between them. Governance networks could also increase public participation and engagement, and thereby contribute to the empowerment of citizens [34]. Thus, there is a need to conduct empirical studies on the accountability of new actor groups such as strategic coordination bodies.

### Accountability in strategic coordination bodies

The term *accountability* refers to both the subject and the object of accounts [35,36]. The subject of an account is the person who is held accountable, whereas the object of an account is that which is accounted for in providing the account, whether it is events, actions, physical objects, or anything else. Esmark [37] specified three dimensions of democratic accountability of a governance network: *inclusion, publicity*, and *responsiveness*; all three should be in place in order for democratic accountability to function.

#### Accountability and inclusion

The first challenge is to identify the “holders” and “holdees” of accountability. The question of inclusion is probably the most fundamental question in democratic theory and practice: Who should be included in a political community (and by implication, who can legitimately be excluded)? The relationship between accountability holders and holdees should not be seen as a relationship inside networks, but rather as a relation between accountability holders (i.e., stakeholders) outside the network and their accountability holdees within the network. It follows that the network should not be considered to be a unified collective with a common set of stakeholders; rather, it should be seen as an array of network members, each with their own set of stakeholders to whom they are accountable [38].

#### Accountability and publicity

The second challenge is about creating sufficient publicity—that is, available information. Perhaps the most persistent critique of network governance is that networks can be closed, inaccessible, and dominated by technocratic discourse. There are at least three issues to consider here, according to Esmark [39]: (1) maintaining records and journals, (2) the role of media, and (3) the extent to which networks conform to a discourse of accountability.

It should be noted here that a great deal of performance information is available about healthcare and social services in Swedish counties and municipalities nowadays. The most authoritative of those systems is called Open Comparisons (OC, or in Swedish, *Öppna Jämförelser*); this system was introduced in the health sector in 2006. The overall objective of OC is to improve the performance of public services; transparency, better opportunities for management control (including accountability), and consumer choice are other objectives. The OC system is based on centrally defined performance indicators that are published annually on the Internet and in written reports; these include indicators that measure structural aspects of the services (e.g., staff competence), processes (e.g., risk prevention), and outcomes (e.g., users’ perceptions of the services). The indicators are based on data from financial accounts, quality records, and user surveys. The total number of performance indicators for municipal social services is more than 350 and there are 15 indicators of the quality of coordinated elder care, such as rehabilitation, the use of (inappropriate) drugs, risk prevention, palliative care, and so forth [40].

#### Accountability and responsiveness

According to Esmark [41], the third challenge is to ensure adequate responsiveness. Accountability implies that accountability holders have some level of control over the accountability holdees. There are two possible sources of accountability: information and sanctions. A presumption of democratic accountability is that unfavorable information leads to the imposition of sanctions on political actors. At least two themes must be considered in this regard: (1) the plurality of possible mandates and sanctions, and (2) the mechanisms through which mandates and sanctions can be imposed —that is, whether the principals are able to punish the agents for wrong and unfaithful actions.

In conclusion, governance networks can be a threat to democracy if they undermine the borderline between state and society. However, according to Esmark [42], this threat can be mitigated if the three dimensions of democratic accountability are met. There are good reasons to assume that the concepts of the model can be used to illustrate accountability challenges in empirical network organizations.

### Methodology

Information about existing coordination bodies for strategic elder care in Sweden was gathered in two steps. First, we conducted an initial telephone survey directed at municipal social service managers responsible for elder care in a randomized stratified sample (based on the official series of municipality numbers), which covered one third of the 290 Swedish municipalities. The reason for using a telephone survey was to make sure that the respondents understood the concept ‘coordination body in elder care’ correctly in relation to our object of study. From this first survey, we obtained information about the existing coordination bodies and their members in the selected municipalities.

Based on this information, we selected 73 coordination bodies dealing with both healthcare and elder care for frail older people in a second survey, which was carried out in 2015 and was directed to all the members – politicians, administrators, and other representatives – in each sampled coordination body. The survey was sent via ordinary mail. The questionnaire contained questions about staffing and representation, working modes, missions and objectives, and perceptions of power and democratic accountability in the operations and in the coordination bodies (aspects identified in previous section). The chosen method of data collection and selection of respondents obviously has weaknesses. It would of course be desirable to also ask similar questions to representatives of potentially critical external reviewers of the coordination bodies, but such design would require a much more resource-demanding setup.

The sample of relevant representatives from the bodies in this survey includes 870 persons, with a response rate of 63 percent (n = 545), and with participation from all 73 sampled coordination bodies for elder care in Sweden. In total, 56 percent (309) of the respondents represent municipalities, and 39 percent (215) represent counties; 2 percent (12) represent local-regional government associations, and 3 percent (13) represent private healthcare providers. The sample contains 31 percent men and 69 percent women. They had in average held current position in their organizations for in average 6 years, and s/he has been involved in a coordination body for in average 4 years.

Among the sample of respondents, 23 percent (127) were politicians, 65 percent (355) were administrators, and 12 percent (62) were social service and health care professionals. Among the politicians, 56 percent (71) were municipal politicians and 44 percent (56) county politicians. Among the managers/administrators, 54 percent (193) come from municipalities, 41 percent represent (146) counties, 2 percent (6) worked in local-regional government associations, and 3 percent (10) come from private organizations. Among professionals, 68 percent (42) work in municipal elder care services, 27 percent (17) in counties health care services, and 5 percent (3) in private services.

## Results

### Background data—The tasks and responsibilities of the coordination bodies

The survey data showed that the geographical responsibilities and policy areas of the coordination bodies varied. Some bodies were responsible for coordinated care throughout the entire county; others were responsible for some part of the county; and others were only responsible for a single geographical municipality, although they held the responsibility for both (municipal/private) social services and (county/private) healthcare. In terms of policy area, some bodies were explicitly oriented toward older people with complex needs, while others were more broadly oriented toward local/regional social and healthcare issues. Regarding their working modes, the survey revealed that the coordination bodies had approximately 5–10 meetings a year.

The survey also showed that the most important task for the coordination bodies was to discuss and solve problems occurring in the local coordinated care system; these discussions were mainly based on local evaluations and on national quality records such as the Open Comparisons. Inspection reports from oversight authorities were also discussed, along with current plans and policy changes in the relevant municipalities and counties (see Table 1).

**Table 1:** **The reported most significant tasks of the coordination bodies—average points** (on a scale from 0 to 3, where 0 means a very low degree of importance, 1 means a fairly low degree of importance, 2 means a fairly high degree of importance, and 3 means a very high degree of importance); (n = 530).


Discuss national quality reports	2.66
Discuss local/regional evaluations	2.82
Discuss remarks of oversight authorities	1.77
Other	1.6


#### I Inclusion

The first theme in the analysis model concerns inclusion—that is, which actors are included as members of the coordination bodies, and which actors may be lacking and should therefore be included. The questions asked were: (1) Which actors are members, and how significant are they in the bodies? (2) Is anyone missing who should be included? (3) How do you perceive the influence of different actors, and your own influence, in the coordination bodies? (4) Which actors contribute most to quality in coordinated care? (5) Who is responsible for a possible lack of quality in coordinated care? According to Esmark [43], the question of inclusion is probably the most fundamental question in democratic theory and practice. Who should be included in a political community and, by implication, who can legitimately be excluded? Table 2 shows the staffing of the coordination bodies.

**Table 2:** **The staffing of the coordination bodies** (percentage of the coordination bodies with members from each category); (n = 513).


Politicians of counties	49
Top politicians of municipalities	28
Politicians of municipal social councils	47
Managers of social services	87
Social service professionals	80
Managers of counties	75
Managers of hospitals	77
Managers of PHC	80
Managers of private service providers	43
Representatives of CSOs	2
Other	27


As indicated in Table 2, no actor group has members in all coordination bodies. There are politically dominated bodies, bodies with managers and other civil servants, and also mixed bodies that include representatives from civil society organizations and private entrepreneurs as well as politicians and civil servants. The actor groups that have members in the greatest number are senior social services managers and their primary health care counterparts. Municipal politicians and county council politicians are members of almost 50 percent of the coordination bodies, and an almost equal proportion of bodies have private entrepreneurs as members. Fairly few coordination bodies have members from civil society organizations (CSOs), which can probably be explained by the fact that there are retirement councils in most municipalities that have existed for a long time, but that are mostly used for general consultation issues concerning the general conditions for retired citizens. Nevertheless, according to the vast majority of respondents, no specific actors are missing that should be included in the coordination bodies of which the respondents are members.

But who has most influence in the coordination bodies? How do the respondents perceive their own influence in the coordination bodies? Moreover, who has the most influence in the daily care of and services for older people? The question here is which actor groups are considered to be most in control of the quality of care and services—that is, which actor group can affect the quality on the user level, whether through resource allocation or by participation in daily operations. (The questions we asked were: What actor group has the most influence within the coordination body? How do you perceive your own and other actor groups’ influence within the coordination body? What actor group has the most influence on the operational services for frail older people in the involved organizations? What actor group should be blamed if the quality of care of the operational services for frail older people is low?) These assessments are shown in Table 3.

**Table 3:** **Perceived influence – How do you perceive your own and other actor groups’ influence within the coordination body? What actor group has the most influence on the operations and on poor quality in operational services? Average points** (on a scale from 0 to 3, where 0 means a very low influence, 1 means a fairly low degree of influence, 2 means a fairly high degree of influence, and 3 means a very high degree of influence).

	Influence within coord. body	Influence on operations	Influence on poor quality

	(n = 533)	(n = 500)	(n = 531)

Top politicians of municipalities	1.74	1.21	1.46
Politicians of municipal social councils	1.95	1.67	1.74
Management of social services	2.2	2.24	2.06
Politicians of counties	1.93	1.64	1.85
Top managers of counties	2.01	2.06	1.76
Managers of hospitals	1.93	2.06	1.85
Managers of PHC	1.96	1.99	1.81
Private providers of social services	1.06	1.26	N.A.
Private providers of PHC	1.38	1.26	N.A.
Operating staff for elderly services	N.A.	N.A.	1.38
Operating staff for hospital care	N.A.	N.A.	1.33
Operating staff for PHC	N.A.	N.A.	1.37
Civil society organizations	1	0.57	N.A.
Other groups	1.5	1.25	0.43
My own influence within coord. body (n = 542)	1.71	N.A.	N.A.

First, we can note that the members themselves, on average, perceive themselves to have a fairly strong influence within their coordination bodies (1.71 on average; there were some minor but not statistically significant differences between the different actor groups). Furthermore, we can note that municipal social service managers is the group of actors that is ascribed to have the most influence within the coordination body, followed by county top managers. These two groups also appear to have the greatest control over the operations. In addition, we asked a question about which actor groups are seen as being the most responsible for a possible *lack* of quality in care and service operations. This question was asked because several other groups may, in fact, be able to create poor quality in daily operations; examples might include social service or healthcare operational personnel. However, the third column in Table 3 shows a similar pattern to those in the former columns. According to the respondents, it is largely the actor groups that are present in the coordination bodies that are responsible for any potential lack of quality. In conclusion, the coordination bodies are generally staffed in a reasonable manner, according to the inclusion criteria on which our model is based.

#### II Publicity

The second theme in the analysis model is that of publicity. A very important issue regarding accountability is, of course, whom the members of the coordination bodies perceive as their principals, and how the reporting was done. The alternative reporting methods specified in the questionnaire were: (a) individual members’ oral reports, (b) individual members’ written reports, (c) joint oral reporting by the coordination body, and (d) joint written reports. Some municipalities draw up so-called collaborative final accounts in which the coordination bodies report their activities; these accounts are similar to other accounting documents that lack a specific group of principals in mind (see Table 4).

**Table 4:** **Reported major modes of providing accounts from coordination bodies to their home organizations—average points** (on a scale from 0 to 3, where 0 means not at all, 1 means a fairly low degree of importance, 2 means a fairly high degree of importance, and 3 means a very high degree of importance); (n = 529).


Oral reports	1.55
Written reports	0.98
Joint oral reports	1.42
Joint written reports	2.13


A very important issue in any accountability arrangement is, of course, who the agents consider to be their principals, or any other kind of legitimate reviewers of their activities. As shown in Table 5, the managers of local social services are considered to be the most important reviewers of what is going on in the coordination bodies. Most of the external groups, such as the media, relatives, and civil society organizations (CSO), are perceived by the members of the coordination bodies as highly passive in their reviews. The same is true for oversight authorities and auditing agencies, which are expected to have independent investigative roles. The actor groups that are perceived as the most important and active reviewers are, consequently, the municipal and county administrations and, to some extent, politicians in social councils and county councils.

**Table 5:** **The reviewers – To what degree do you perceive the following actors as reviewers of the coordination body? — average points** (on a scale from 0 to 3, where 0 means very limited, 1 means a fairly low degree, 2 means a fairly high degree, and 3 means a very high degree); (n = 529).


Top politicians of municipalities	1.07
Politicians of municipal social councils	1.48
Managers of social services	2.07
County politicians	1.52
County top management	1.68
Managers of hospitals	1.69
Managers of PHC	1.66
CSO representatives	0.66
Media	0.62
Audit agencies	0.93
Healthcare/social inspectorates	0.83
Relatives	0.48


#### III Responsiveness

The third theme, responsiveness, refers to what mandate the members of the coordination bodies perceive themselves as having from their home organizations. All members of the coordination bodies are supposed to have mandates from their “home organization,” either as a politician, civil servant, or other representative. As shown in Table 6, the different respondents perceive their mandates as being fairly clear and strong. The group that represents private healthcare/service providers has a lower scoring here. One interpretation may be that this group experiences uncertainty regarding what they are really expected to do and achieve within the highly political context of the coordination bodies. However, our data does not provide much information about what kind of sanctions could be imposed on the members of the coordination bodies.

**Table 6:** **Members’ perceptions of their mandates — average points** (on a scale from 0 to 3, where 0 means a very weak mandate, 1 means a fairly weak mandate, 2 means a fairly strong mandate, and 3 means a very strong mandate); (n = 531).


Municipal representative	2.47
County representative	2.34
Municipal and county	2.5
Private providers	2.0
Politicians	2.3
Managers	2.45
Professionals	2.32


## Discussion

The survey provided good empirical answers to the first question on how the coordination bodies are staffed and how the members perceive their tasks and responsibilities. However, there are good reasons to take a somewhat deeper look at the second question with different aspects of accountability. The first challenge according to the model developed by Esmark [44] was: How is the inclusion challenge handled in the coordination bodies? Are there any weaknesses and shortcomings in these respects? According to the respondents, it is largely the groups that have members in the collaboration bodies that are also perceived as being responsible for the quality – and also for the possible lack of quality of care. Therefore, the coordination bodies seem to be staffed in a reasonable manner, according to the inclusion criteria on which our model is based.

The second challenge is about publicity and the availability of information on quality of care? Are there any weaknesses and shortcomings here? A great deal of information about quality of care is now available through the Open Comparisons, which were introduced in previous sections. However, external stakeholders such as the media, civil society organizations, oversight authorities, and auditing agencies seem to be perceived as highly passive in their reviewing roles. The stakeholders that are perceived as the most active reviewers are, interestingly, the municipal and county municipal administrations and, to some extent, the politicians in the social welfare boards and the county councils. This is interesting because these actor groups probably focus more on formal aspects of the coordination bodies than on more critical and sensitive aspects of the care of frail older people. Here it would, of course, be desirable to obtain assessments from potential external critical reviewers, but a prudent conclusion would be that this situation could be problematic from a democratic point of view, since most of the reviewers are more or less internal.

The third challenge is about how the responsiveness of the coordination bodies is handled? Are there any weaknesses and shortcomings here? Although the politicians and civil servants feel that they have a clear mandate from their home organizations—which they probably perceive as the principals that they must report back to—it is not certain that the *citizens* clearly know to whom they should direct their criticisms when coordinated care of the elderly is failing. According to Provan and Milward’s [45] seminal study of networks for the mentally disabled, presented earlier, network effectiveness is enhanced through centralization and when mechanisms of external control are direct and not fragmented. If these conditions are also relevant in a Scandinavian welfare context, there may be potential for quality improvement in Swedish services for frail older people, and there may also be potential for improvement in terms of democratic accountability.

Finally, we address the last questions in the analysis, namely: What are the most challenging issues for these coordination bodies from a democratic point of view? Are there any major democracy deficits? How could these be reduced? Behind these questions rests, of course, also the ‘so what’ question: What importance do these issues have for frail older people who often “fall through the cracks” in the care system?

The limited presence of external scrutiny and the presence of some uncertainty regarding who should be held responsible may indicate a high degree of trust between potential principals and potential agents; this would not necessarily constitute a major democracy problem. However, governmental investigations, ambitious reform attempts, and attempts to measure the quality of coordinated care have indicated that many shortcomings still exist in the care of frail older people in Sweden. As indicated in the introduction of the paper, most reforms have been directed to improve collaboration between organizations and professions and also to introduce better coordinated working processes among the staff. But, as indicated by Provan and Milward [46], centralized external control seems to have an extensive impact on effectiveness and quality of care for vulnerable people. A more developed public debate on who holds the actual responsibility for care and services, together with an effort to spreading performance information on coordinated care, could increase the interest in the democratic governance and accountability of healthcare and social care among important stakeholder groups, such as civil society organizations, the media, and external audit and supervisory authorities. This would also make it clearer for fragile older people and their relatives where they should address their criticism when the coordinated care fails. If the coordination bodies became more visible, more known and more often held responsible for deficiencies in the elderly care system, the pressure on improvements would probably increase and it would be more difficult to blame the shortcomings on actors with less power. As stated by Olsen [47], perhaps “the sleeping bear” should wake up. This means that the issue of democratic accountability is not only an issue for citizens in general but could also be highly relevant for frail older people who often “fall through the cracks” in the welfare system.

## Conclusions

The aim of the paper was to analyze coordination bodies as important actors in integrated care, and especially to investigate how the members of these governance networks perceive their own influence and how they are held accountable by their principals. We fulfilled this aim by investigating how the coordination bodies are staffed and by asking their members about their tasks and responsibilities. Moreover, we investigated members’ perceived influence and their perception of how they are held accountable by their principals. Furthermore, we attempted to explore the most challenging issues facing these coordination bodies from a democratic point of view.

This study has potential policy implications. The establishment of successful collaborative and coordination arrangements that help to meet the needs of vulnerable groups is undoubtedly one of the greatest challenges facing the modern welfare society. The relationship between power and accountability in healthcare and in the municipal social services is a fundamental democratic issue that should be discussed further among citizens, politicians, and civil servants, and the investigation that is presented here can contribute to this discussion. One outcome of this discussion may be that citizens will increase their demands that politicians reclaim their political influence over the social service and healthcare agencies.

The contribution of the paper to the current understanding of democratic governance is mainly empirical. This paper builds on the model of accountability in networks that was developed by Esmark [48] and finds that model not only useful for investigating if and how governance networks can be perceived as democratic, but also helpful for identifying possible problematic areas in accountability arrangements. Even though we have presented quantitative indicators of perceived accountability at actor group level, we mainly wanted to test and illustrate the model rather than trying to explain our measurements at the coordination body level. More empirical research on accountability in governance networks is necessary. Case studies could help us to include more potential reviewers and critics in the analyses in order to understand the dynamics in local governance networks. It would also be interesting to compare how different governance networks work from a democratic and accountability perspective in different countries and within different policy areas.

## Author Information

Staffan Johansson is Professor in Social Work at the Department of Social Work, University of Gothenburg. Staffan Johansson’s research is about human service organizations, including policy implementation, issues around collaboration and coordination the services for disadvantaged citizens, and also the roles of civil society organization in the welfare state.

Andreas Liljegren is Associate Professor in Social Work at the Department of Social Work, University of Gothenburg. Andreas Liljegren’s research is on the relation between professions, laypersons and organizations, and also on workload in street level bureaucracies. His research also covers collaboration between the social and the health sector.
